# Cyanobacterial Mats in Calcite-Precipitating Serpentinite-Hosted Alkaline Springs of the Voltri Massif, Italy

**DOI:** 10.3390/microorganisms9010062

**Published:** 2020-12-29

**Authors:** Aysha Kamran, Kathrin Sauter, Andreas Reimer, Theresa Wacker, Joachim Reitner, Michael Hoppert

**Affiliations:** 1 Institute of Microbiology and Genetics, University of Göttingen, Grisebachstraße 8, 37077 Göttingen, Germany; akamran@gwdg.de (A.K.); kathrin.sauter@stud.uni-goettingen.de (K.S.); tw492@exeter.ac.uk (T.W.); 2 Göttingen Centre of Geosciences, Georg-August-University Göttingen, 37077 Göttingen, Germany; areimer@gwdg.de (A.R.); jreitne@gwdg.de (J.R.); 3 Medical Research Council Centre for Medical Mycology (MRC CMM), University of Exeter, Geoffrey Pope Building, Stocker Road, Exeter EX4 4QD, UK

**Keywords:** microbial diversity, cyanobacteria, serpentinite, alkaliphilic, carbonate

## Abstract

(1) Background: Microbial communities in terrestrial, calcifying high-alkaline springs are not well understood. In this study, we investigate the structure and composition of microbial mats in ultrabasic (pH 10–12) serpentinite springs of the Voltri Massif (Italy). (2) Methods: Along with analysis of chemical and mineralogical parameters, environmental DNA was extracted and subjected to analysis of microbial communities based upon next-generation sequencing. (3) Results: Mineral precipitation and microbialite formation occurred, along with mat formation. Analysis of the serpentinite spring microbial community, based on Illumina sequencing of 16S rRNA amplicons, point to the relevance of alkaliphilic cyanobacteria, colonizing carbonate buildups. Cyanobacterial groups accounted for up to 45% of all retrieved sequences; 3–4 taxa were dominant, belonging to the filamentous groups of Leptolyngbyaceae, Oscillatoriales, and Pseudanabaenaceae. The cyanobacterial community found at these sites is clearly distinct from creek water sediment, highlighting their specific adaptation to these environments.

## 1. Introduction

Carbonates, formed both abiogenically or biogenically, are among the most abundant minerals, and are essential in the long-term global carbon cycle [1]. As dissolved ions, they are the most important parameters for ocean alkalinity [2]. Precipitation of a variety of carbonate mineral phases involving microorganisms has been reported under various environmental conditions [3,4,5,6]. Directed mineral precipitation (biomineralization) occurs through active biological control by an organism (mainly by eukaryotic algae and metazoans [7]). Cyanobacteria, in particular, are considered to be involved in carbonate precipitation within oceans, lakes, springs, caves, and even soil [8]. Active uptake of CO_2_, as HCO_3_^−^ from an aqueous solution, results in a net increase in OH^−^ ion concentration and pH increase in the environment. In the cell, HCO_3_^−^ is converted to CO_2_ (for carbon dioxide fixation by the ribulose bisphosphate carboxylase enzyme) and a hydroxide ion, resulting in a shift to alkaline pH outside the cell. At elevated pH, the equilibrium between carbonic acid, hydrogen carbonate, and bicarbonate is shifted to the deprotonated carbonate ion forms. Metal cations, mainly calcium and magnesium ions, combine with those ions and form precipitates of Ca/Mg-carbonates. Respiratory processes, however, invert the pH shift, and precipitated CaCO_3_ will be partly redissolved; by oxidation of organic compounds, CO_2_ is released. In aqueous solution, dissolved CO_2_ forms the weak acid H_2_CO_3_, resulting again in the formation of H^+^ and HCO_3_^−^ upon dissociation, which decreases pH. When CO_2_ fixation and respiration are balanced against each other, considerable net carbonate precipitation may not be expected. In calcium-oversaturated systems, carbonate precipitation by biogenic shift of pH is negligible, but chemical precipitation becomes a challenge for microbial communities, due to rapid encasement by the mineral. As a countermeasure, (cyanobacterial) sheaths and/or capsular material precipitate calcium carbonate by attracting calcium ions to acidic groups of their exopolysaccharides (the carboxylic acid groups are mainly deprotonated at high pH), and mineral precipitation is directed to sheaths or capsules [5,9]. In many carbonate-precipitating alkaline water bodies, cyanobacteria and other organisms are involved in formation of carbonate buildups [4,5,10,11,12]. Because of the overall high pH, photosynthesis-driven pH shifts may be negligible, and carbonate precipitation at any nucleation site of a cell surface is inevitable. Serpentinization-driven high-alkaline springs may be considered as model systems for carbonate precipitation under participation of microorganisms. Frequently, travertine-like carbonate buildups form at the spring seeps [13,14,15]. The pH values are mostly higher (11–12) than in alkaline lakes (8–10) [16]. Because of low sodium concentrations in the alkaline springs, the environment does not correspond to soda lake conditions [15,16,17].

A number of these high-alkaline springs are located in the massifs of Voltri (Gruppo di Voltri), near Genoa. The massifs originate from Upper Jurassic oceanic crust belonging to the Piedmont–Ligurian Ocean of the Western Alpine ocean system [18,19]. Due to tectonic processes during the Eocene, ultramafic (peridotitic) rocks of the oceanic crust were subjected to metamorphosis, resulting in, e.g., eclogite and amphibolite facies [20]. After uplifting, the influence of meteoric water, and putatively seawater, under certain pressure and temperature conditions lead to conversion of olivine, pyroxenes, and amphiboles in the ultramafic rocks. As a result of this metamorphic process, serpentinite minerals are formed. The underlying process, termed serpentinization, comprises multiple reactions, involving hydrolyzation and oxidation of the starting minerals. 

As one important reaction in this process, the olivine constituent forsterite is converted with water to serpentine (the name-giving mineral of the process) and brucite:

2 Mg_2_SiO_4_ + 3 H_2_O → Mg_3_Si_2_O_5_(OH)_4_ + Mg(OH)_2_ (1)

In the following, just some aspects about the complex geochemical processes are briefly mentioned, because their reaction products are potentially relevant for microbial processes. 

The olivine constituent fayalite is oxidized to magnetite, which also results in release of molecular hydrogen:

3 Fe_2_SiO_4_ + 2 H_2_O → 2 Fe_3_O_4_ + 3 SiO_2_ + 2 H_2,_ (2)

Molecular hydrogen reduces, in presence of catalysts (e.g., awaruite), carbon monoxide and carbon dioxide to short-chain hydrocarbons, most simply methane (e.g., [21]):

4 H_2_ + CO_2_ → CH_4_ + 2 H_2_O (3)

Dissolution of forsterite and diopside produces calcium and hydroxide ions [22,23]:

4 Mg_2_SiO_4_ + CaMgSi_2_O_6_ + 7 H_2_O → 3 Mg_3_Si_2_O_5_(OH)_4_ + Ca^2+^_(aq)_ + 2 OH^−^_(aq)_ (4)

Alkaline fluids pass through faults and fractures from their sources to the surface, where they emerge in springs. A microbial community of this serpentinizing environment is thought to be involved in the recycling of the released hydrogen and methane gas. Microorganisms from these communities have been recovered from borehole depths of up to 130 m below ground [24,25]. These subsurface microbial processes comprise the oxidation of hydrogen by hydrogenotrophic methanogenic archaea (resulting in the formation of methane), or by acetogenic bacteria (formation of acetate). Acetate may be utilized by acetotrophic methanogens; methane may be oxidized anaerobically in the deep subsurface, as long as appropriate electron acceptors (generally sulfate) are present. Analysis of the microbial community of the pristine spring water revealed the presence of hydrogen- and methane-consuming microbiota [15]. The anaerobic subsurface processes and aerobic respiration at the surface produce CO_3_^2−^ and HCO_3_^−^ ions, which precipitate with divalent cations (Ca^2+^, Mg^2+^) to form carbonate minerals (microbial carbonation [26]).

It is obvious, however, that small pools formed by the high-alkaline fluids are also exposed to the microbial communities of surrounding terrestrial and aquatic sources. The alkalitolerant organisms of these communities may be potential colonizers for the highly alkaline fluids. Our study focuses on presence and abundance of these organisms colonizing pools just next to the outflows of the high-alkaline springs. Inevitably, these organisms must cope with the rapid precipitation of carbonates in this environment.

## 2. Materials and Methods 

### 2.1. Sampling

The samples were taken from Voltri Massif (Liguria, Italy), from two different locations at approximately 44°25′27.4”N, 8°39′31.4”E (Torrente Lerone, LER) and 44°26′43.3”N, 8°46′43.0”E (Torrente Branega, BR). Several subsamples (LER subsamples: VE01-02.B, VE01-03.C, VE02-01.B, VE02-02.B, and BR subsamples: V04-01-BR1-1, V04-02-BR1-2a, and V04-03-BR1-3) were taken from carbonate crusts or bottom sediments of ponds. All samples were collected with sterile tools (bailer, scalpels, tubes). All samples were kept in a mobile freezing unit at −20 °C, immediately after collecting, and were kept frozen until further processing.

For petrographic analysis, pieces of carbonate buildups and rocks were sampled with spatulas or hammer and chisel. Ophicalcite samples were taken from outcropping rocks (44°11′29.1”N, 9°35′42.9”E; NE of Bonassola/La Spezia).

### 2.2. Hydrochemical Analysis

Physicochemical parameters of spring and pool waters were measured in field sites using a WTW Multi 3430 device equipped with a WTW Tetracon 925 conductivity probe, a Schott PT61 redox electrode, and a WTW Sentix 940 electrode for temperature and pH measurements (Xylem, Rye Brook, NY, USA), calibrated against standard pH buffers 7.010, 10.010, and 12.000 (HI6007, HI6010, and HI6012, Hanna Instruments, Woonsocket, RI, USA).

Spring and pool waters were collected without headspace in Schott Duran glass bottles (Schott, Mainz, Germany) and polyethylene bottles of varying size and were considered for the determination of anions, cations, nutrients, and total alkalinity (TA), as well as dissolved inorganic carbon (DIC). For cation analysis, a 50 mL aliquot was filtered through 0.7 µm pore membrane filters and acidified with 100 µL HNO_3_ (Suprapur, Merck, Darmstadt, Germany). For determination of total sulfide (ΣH_2_S), aliquots were fixed with Zn-acetate. Samples were stored refrigerated in the dark until further processing. Total alkalinity was determined by acid–base titration immediately after sampling, using a hand-held titration device and 1.6 N H_2_SO_4_ cartridges as titrant (Hach Lange GmbH, Düsseldorf, Germany).

Main cations (Li^+^, Na^+^, K^+^, Mg^2+^, Ca^2+^, Sr^2+^) and anions (F^−^, Cl^−^, Br^−^, NO_3_^−^, SO_4_^2−^) were analyzed by ion chromatography with non-suppressed and suppressed conductivity detection (Metrohm 820 IC, Metrohm 883 Basic IC; Metrohm, Herisau), respectively. Concentrations of NH_4+_, NO_2−_, PO_4_^3−^, ΣH_2_S, and dissolved silica were determined photometrically according to [27], using an SI Analytics Uviline 9400 spectrophotometer. Dissolved inorganic carbon was determined with a TOC-L CPH analyzer (Shimadzu, Kyoto, Japan). 

The PHREEQC software (version 3.5.0, 2019 [28]), using the phreeqc.dat and wateqf4.dat databases, was applied for calculation of ion activities, pCO_2_ (partial pressure of CO_2_) of samples, and mineral saturation states. Saturation is given as SI = log (IAP/KSo), where IAP denotes ion activity product and KSo solubility product of the mineral phase.

### 2.3. Microscopic Analysis and Petrography 

Petrographic thin sections of approximately 30–60 µm in thickness were essentially performed according to [29]. Light microscopic analysis of sections was conducted with a Zeiss SteREO Discovery V8 stereomicroscope (transmitted and reflected light) linked to an AxioCam MRc 5-megapixel camera (Zeiss, Göttingen, Germany).

Light microscopy of cyanobacteria was performed using a Motic BA310E microscope (Motic GmbH, Wetzlar, Germany) with phase contrast optics and equipped with a Color view III camera (Motic GmbH). Micrographs were processed using the cell D image software (Motic GmbH). Raman microscopy in conjunction with Raman spectrometric analysis of petrographic sections was performed as described [30].

For scanning electron microscopy, samples of about 1 cm^3^ were dehydrated in an ascending ethanol series (15% to 99%), mounted on SEM sample holders, and sputtered with Au–Pd (7.3 nm for 120 s). Field emission scanning electron microscopy (FE-SEM) was performed using a Carl Zeiss LEO 1530 Gemini system (Zeiss, Oberkochen, Germany).

Stable isotope measurements (^12^C, ^13^C, ^16^O, ^18^O) on carbonates were conducted at the Department of Isotope Geology (Göttingen Centre of Geosciences) using a Thermo KIEL VI/Finnigan Delta+ gas mass spectrometer (Thermo Fisher Scientific, Waltham, Mass., USA). A total of 100 mg of powdered whole rock material was analyzed, respectively. Data are expressed as δ value relative to Vienna Pee Dee Belemnite (V-PDB). Values reported here have an analytical error of <0.2%.

### 2.4. DNA Extraction

DNA was extracted from 0.35 g of a single sample with the Power Soil DNA isolation kit (Qiagen, Hilden, Germany), following the manufacturers protocol. After extraction, agarose gel electrophoresis was used to estimate the approximate size distribution of the extracted DNA from each sample (1 kb DNA size standard ladder, 1%, *w/v*, agarose; Thermo Fisher Scientific). Concentration of DNA from each sample was estimated in a Nanodrop spectrophotometer (Thermo Fisher Scientific). Polymerase chain reaction (PCR)-based amplification of the V3 and V4 region of the bacterial 16S rRNA was performed in triplicates from each sample by thermal cycler processing. For the appropriateness of the amplicons for Illumina MiSeq sequencing (Illumina, San Diego, CA, USA), primers were additionally linked to the overhang adapter sequences (16S amplicon PCR forward primer = 5′-TCG TCG GCA GCG TCA GAT GTG TAT AAG AGA CAG CCT ACG GGN GGC WGC WGC AG-3′; 16S amplicon PCR reverse primer = 5′-GTC TCG TGG GCT CGG AGA TGT GTA TAA GAG ACA GGA CTA CHV GGG TAT CTA ATC C-3′; adaptor sequences according to the manufacturers note, primers for bacterial 16S rRNA genes according to [31]). Master Mix was prepared according to [31], with minor modifications: MgCl_2_ 25 mM (0.15 µL), DMSO 5% (2.5 µL), 5× Phusion GC Buffer (10 µL), dNTPs 10 mM (1µL), reverse and forward primer (1:10 dilution), 2 U/µL Phusion HF DNA polymerase (0.5 µL), and template DNA 25 ng (1 µL) made up to the final volume of 50 µL with double-distilled nuclease-free water (Phusion GC buffer and polymerase: Thermofisher Scientific). The following thermal cycler program was used: initial denaturation at 98 °C for 1 min, 25 cycles at 98 °C for 45 s, 60 °C for 45 s, 72 °C for 30 s, and, finally, one cycle at 72 °C for 5 min. 

For gel electrophoresis, 1 µL HDGreen stain (INTAS, Göttingen, Germany) was mixed with 3 µL of the PCR product solution and separated with a 1.3% (*w/v*) agarose gel in a Tris-acetate-EDTA-buffer (TAE)-system/ [32], while a 1 kb DNA ladder (Thermofisher Scientific) was used as size standard. A NanoDrop photometer (Thermofisher Scientific) was used to quantify the DNA in the reaction mixture. The GeneRead Size Selection Kit (Qiagen, Venlo, The Netherlands) was used for the selection of target amplicons (~500 bp). An Illumina MiSeq desktop sequencer (Illumina, San Diego, CA) was used for the sequencing, employing the MiSeq Reagent Kit v3 (2 × 300 bp paired-end reads; Illumina).

### 2.5. Sequence Data Processing

Paired-end sequencing of amplicons and processing of sequencing data were carried out in the Göttingen Genomics Laboratory (Göttingen, Germany). The software FastQC v0.11.8 [33] was used to check and verify the paired-end reads. After merging the sequences, short reads (less than 305 base pairs) and those with unidentifiable bases were eliminated with PANDAseq v2.11 [34] using the PEAR algorithm v0.9.8 [35]. Forward and reverse primer sequences were eliminated using cutadapt v1.15 [36]. The amplicon sequences were processed with QIIME 1.9.1 [37]. The sequences were dereplicated and verified for chimeric sequences (de novo). The sequences were clustered into operational taxonomic units (OTUs) with 97% identity. The taxonomic classification of the OTU sequences was also carried out with QIIME 1.9.1 against the SILVA database 132 using the assignment method [38], with chloroplasts, extrinsic domains, and unclassified OTUs being eliminated from the dataset. Sample comparisons were performed at the same surveying effort, utilizing the lowest number of sequences by random subsampling. All sequences are available via the Biosample database of the NCBI (National Center for Biotechnology Information, Bethesda, MD, USA) under Bioproject accession No. PRJNA685937.

Results were displayed as bar charts based upon the summarized OTU tables. Alpha diversity was expressed as rarefaction curve (number of observed species plotted versus number of sequences retrieved from a sample), as a function of the QIIME pipeline. To visualize the microbial composition between samples by principal coordinates analysis (PCoA), a square matrix expressing the dissimilarity between every pair of samples [39] was calculated in QIIME and rendered by EMPeror (integrated with QIIME [40]) for graphic visualization. Venn Diagrams were plotted based upon the BioVenn application [41].

## 3. Results

### 3.1. Hydrochemistry, Carbonate Fabrics and Isotopic Record

**Figure 1:**
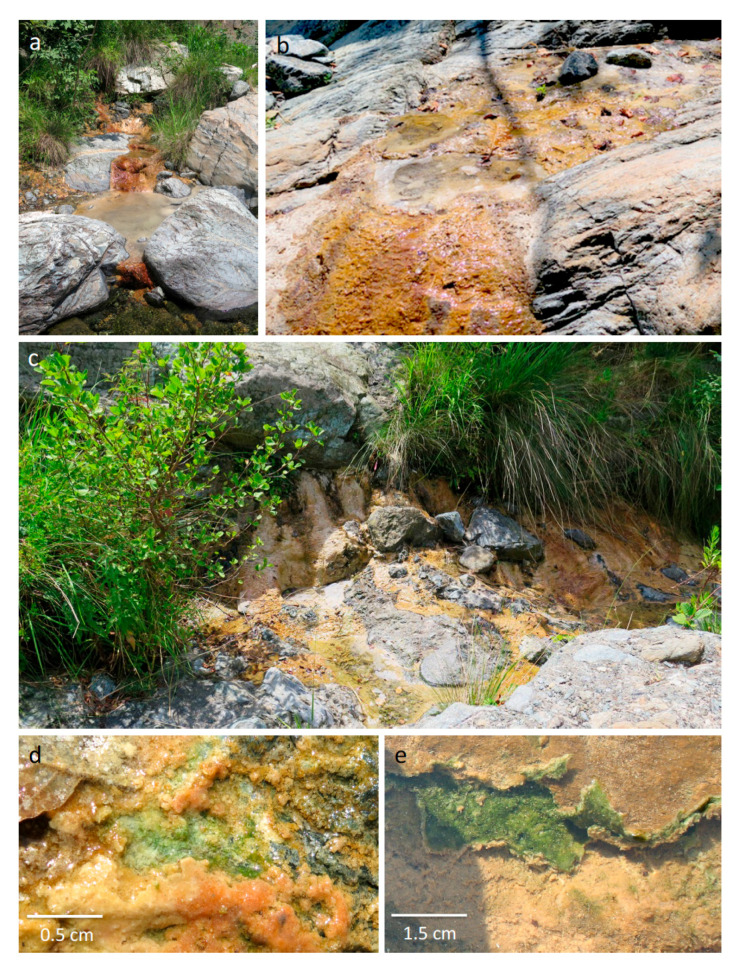
Cyanobacterial biofilms in sampling sites: (**a**,**b**) Torrente Leorne and (**c**) Torrente Branega alkaline pools. (**d**,**e**) Thin greenish layers, dominated by cyanobacteria, are situated below calcitic crusts.

All spring water bodies in the Torrente Branega/Torrente Lerone of the Voltri Massif area show extremely high pH values, mostly above 11 (Table 1, Table S1), and exhibit low total alkalinity (on average 1.75 meq L^−1^ for Torrente Lerone sites and 2.5 meq L^−1^ for Torrente Branega sites). All are oversaturated with respect to calcium carbonate. In both sites, dissolved inorganic carbon is low (1.2–1.3 mg L^−1^). The redox potentials are considerably lower than in creek water, in particular in all samples taken from the Torrente Lerone sites. Generally, concentration of macroelements relevant for microbial growth (nitrate/ammonium, phosphate) are low, i.e., the sites are highly alkaline, calcite-precipitating, oligotrophic water bodies. At all springs, carbonate buildups are formed. The carbonates frequently form cascade-shaped crusts at sites where spring water trickles over rock surfaces (Figure 1a,b). Small pools form in depressions in or between rocks; they are calm water bodies with no significant currents. Pool water appears clear; the precipitated calcite particles form a sediment body on the bottom of the pools. Samples were all taken from more or less consolidated calcitic crusts and the sediment of the high-alkaline pools (Figure 1a–c). Figure 1d,e depict the sampling sites and typical features of representative samples.

**Figure 2:**
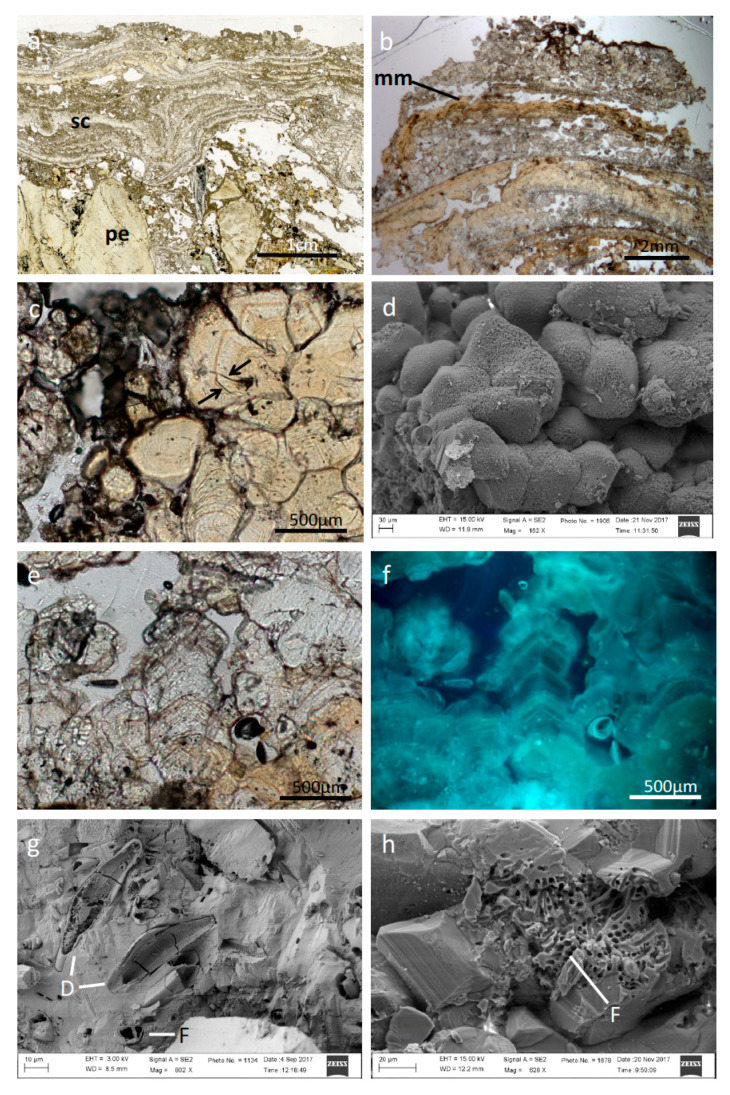
Microstructure of representative carbonate buildups from Torrente Lerone: (**a**) Travertine-like spring carbonate buildups: stromatolitic crusts (sc) overgrowing ultramafic rock pebbles (pe). (**b**) Detailed view of a stromatolitic crust exhibiting mineralized microbial mats (mm, light yellow crusts) alternating with grey calcites and micritic portions. (**c**) Magnified view of the light-yellow anhedral calcites showing entrapped microbial remains (arrows). (**d**) Field emission electron microscopic (FE-SEM) view of the light-yellow calcites. (**e**) Grey-white scalenohedral calcite with growth laminae. (**f**) Same specimen as (**e**) under UV-fluorescence excitation. Blue fluorescence is caused by intracrystalline organic matter. (**g**) FE-SEM view of the grey-white calcites. Frustules of pennate diatoms (D) and holes as remains of microbial filaments (cross sections; F) are visible. (**h**) FE-SEM view of the light-yellow calcites with remains of filamentous microbes (F).

Thin sections of consolidated travertine-like carbonates, perpendicular to the surface (cf. Figure 2a,b) frequently show a stromatolitic fabric. The structure is characterized by 1–2 mm light-yellow calcite crusts, which alternate with light- to dark-grey calcites and micritic areas (Figure 2b–d). The carbonate buildups exhibit a multitude of open pores, characteristic for a fenestral (birdseye) fabric. Calcites contain diatom frustules and other inclusions of putatively microbial origin, like tiny filamentous structures (Figure 2c,g,h). UV excitation reveals various intensities of blue fluorescence, indicative of the presence of organic material (Figure 2e,f; cf. [42]. Generally, Raman spectroscopy (not depicted) showed only low organic contents. Occasionally pyrite crystals are associated with the calcites, which show high contents of organic carbon.

**Figure 3:**
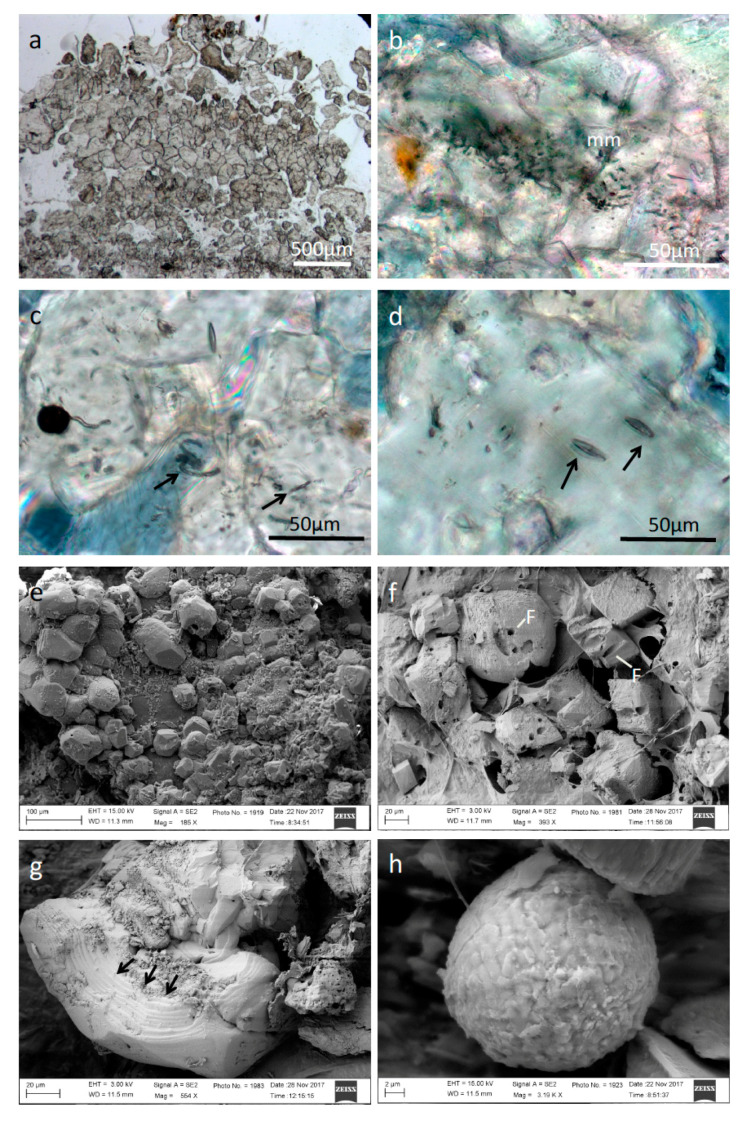
Microstructure of representative carbonate buildups from Torrente Branega: (**a**) Aggregates of grey calcites. Stromatolitic crusts are just weakly developed. (**b**) Microbial mat remains (mm) entrapped in calcites. (**c**) Filamentous microbial remains (arrows) within calcite crystals. (**d**) Pennate diatoms entrapped within calcite (arrows). (**e**) FE-SEM picture of newly formed anhedral calcite crystals. (**f**) FE-SEM picture of nearly euhedral calcite crystals exhibiting holes of formerly filamentous microbes and remains. The crystals are covered by dehydrated exopolymeric substances (EPS). (**g**) FE-SEM picture of an anhedral calcite crystal showing “stromatolitic” growth pattern (arrows). (**h**) FE-SEM picture of a framboidal pyrite globule associated with calcites.

In samples from Torrente Branega (Figure 3), stromatolitic crusts are not clearly developed (Figure 3a); the partly anhedral shape of the calcite crystals, however, corresponds to the observations for specimens from Torrente Lerone sites. In the Branega carbonates, entrapped microbial cells are more abundant (Figure 3a–d); pennate diatoms and filamentous microbial remains are observable. The open pore calcitic carbonate system also exhibits fenestral fabric.

In order to gain more insight into the origin of the carbonate constituents, stable δ^13^C and δ^18^O isotopes of carbonate buildups from Torrente Lerone and Torrente Branega, as well as ophicalcites from a nearby abandoned quarry, were analyzed. In Torrente Lerone carbonates, the δ^13^C_CaCO__3_ values ranged from −24‰ to −11‰; δ^18^O_CaCO__3_ values ranged from −19‰ to–9‰. Most negative values were measured for travertine-like carbonates close to the spring site; with increasing distance from the spring, the isotopic mixtures became less depleted.

In the Torrente Branega site, data differ from the Lerone locality. The measured δ^13^C values were close to 0‰, up to +0.6‰. δ^18^O values ranged between −20‰ and −26‰. For samples at different distances to the spring, no trend was visible. These data exhibit similarity with the cemented carbonate fractures within so-called ophicalcites NE of Bonassola/La Spezia [43]; in many active and abandoned quarries, the serpentinized ultramafic rocks are exposed. They are characterized by abundant carbonate fractures due to strong hydrofracturing during serpentinization. Samples taken from these ophicalcites showed δ^13^C values around +2% and δ^18^O values between −8‰ and −16‰.

### 3.2. Microbial Diversity

**Figure 4:**
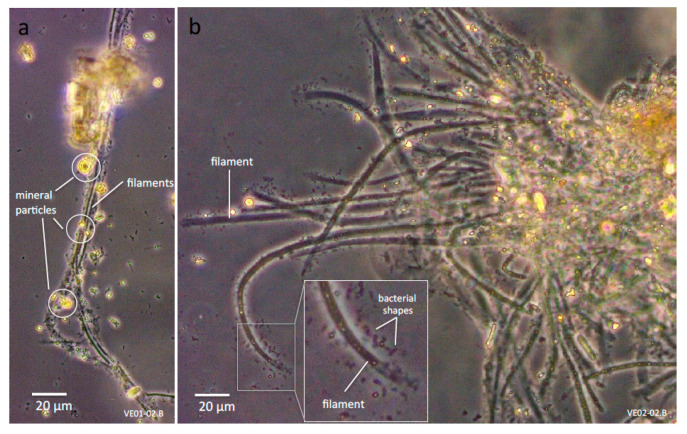
Representative samples depicting filamentous cyanobacterial morphotypes intermixed with calcitic precipitates: (**a**) Sample VE01-02.B; mineral particles attached along cyanobacterial filaments. (**b**) Sample VE02-02.B; multiple bacterial morphotypes (inset) adjacent to a filament.

Green layers of photosynthetic organisms were located below the partially consolidated thin crusts of white and brown precipitates of calcite (Figure 1d,e). Light microscopy reveals bundles of filaments, appearing similar to cyanobacterial morphotypes (Figure 4a), but also empty sheaths of cyanobacterial cells, with small rod-like bacterial morphotypes attached to these sheaths. Mineral particles are attached to these filaments, but do not form tight crusts (Figure 4b).

**Figure 5:**
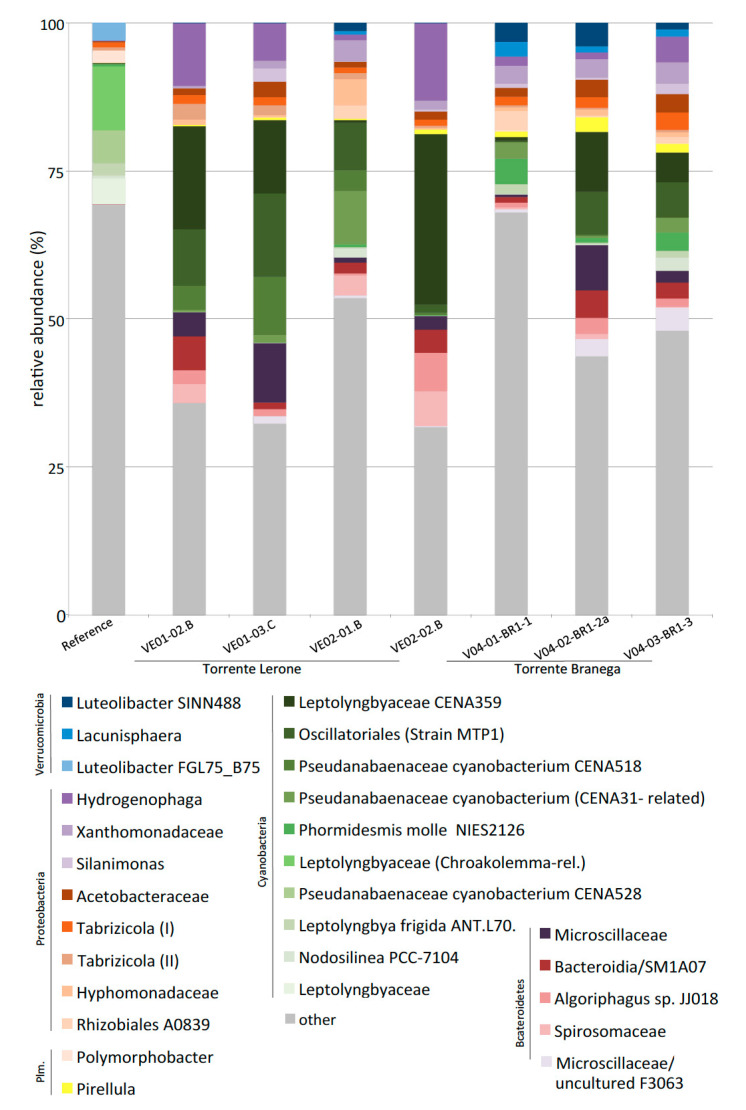
Bar charts showing the most abundant bacterial groups retrieved from all sites (reference: Table 01. VE02, Torrente Lerone spring site; V04, Torrente Branega spring sites).

**Figure 6:**
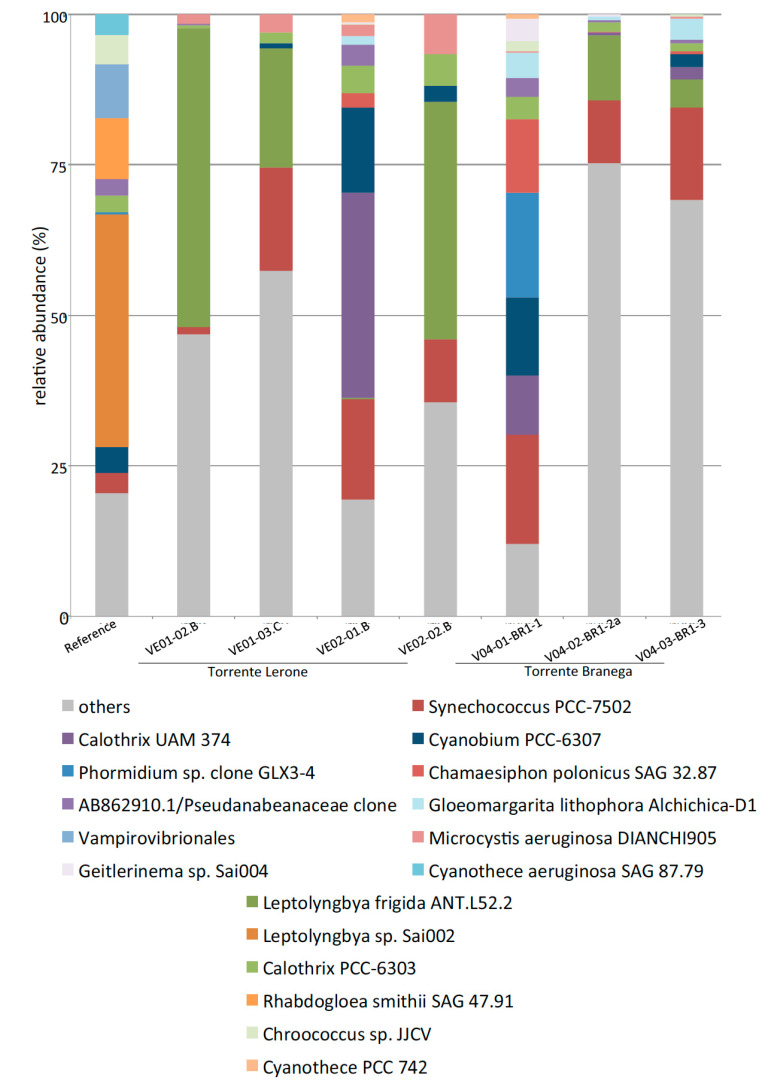
Bar charts showing the minor cyanobacterial groups retrieved from all sites (reference: Torrente Branega creek water sediment; VE01, VE02, Torrente Lerone spring sites; V04, Torrente Branega spring sites).

The operational taxonomic units (OTUs) above 2% relative abundance, at least in one of the samples, is shown in Figure 5. Taxa of less-abundant cyanobacteria, not shown in Figure 5, are depicted in Figure 6. Rarefaction curves for all OTUs (Figure S2) do not reach a plateau, but still show considerable differences in expected species numbers per sample; these are considerably higher in the (non-alkaline) creek water sample (reference), when compared to samples from alkaline pools. Cyanobacteria reach up to 45% of all retrieved OTUs in some samples, with 24% +/−5% in Torrente Branega samples and 38% +/−5% in Torrente Lerone samples.

**Figure 7:**
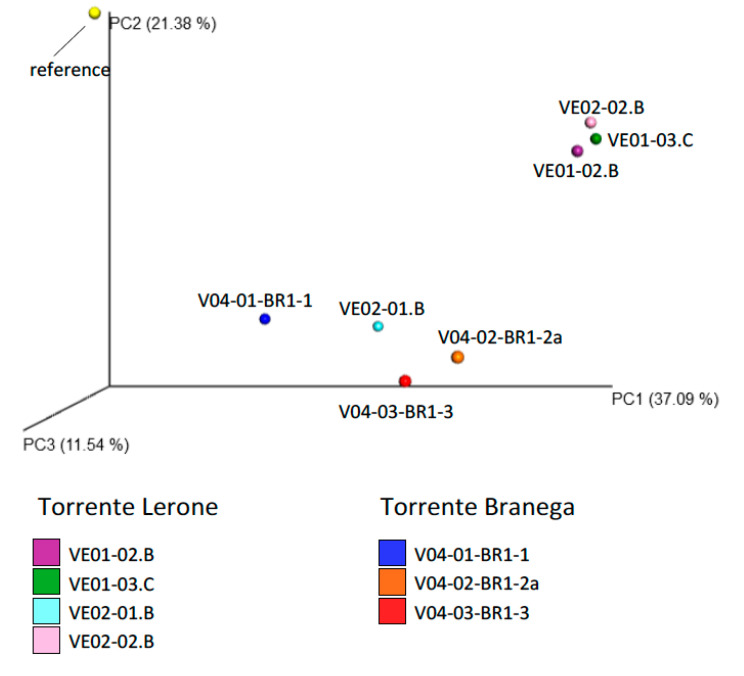
Unweighted principal coordinates analysis (PCoA) plot, depicting all samples as indicated.

The weighted (Figure 7) and unweighted (not depicted) PCoA plots show that the beta diversities from spring sites form two separate clusters. All Torrente Branega samples form a cluster, along with one of the Torrente Lerone samples. Apparently the relative abundance of the dominant taxa of sample VE02-01.B is similar to the respective community of Torrente Branega samples (cf. Figure 5), though environmental data (cf. Table S1) of the VE02-01.B sampling site do not greatly differ from those of the other Torrente Lerone sites. All other samples from Torrente Lerone form a separate, broader cluster. All communities are clearly distinct from creek water sediment, taken from Torrente Branega as a reference.

**Figure 8:**
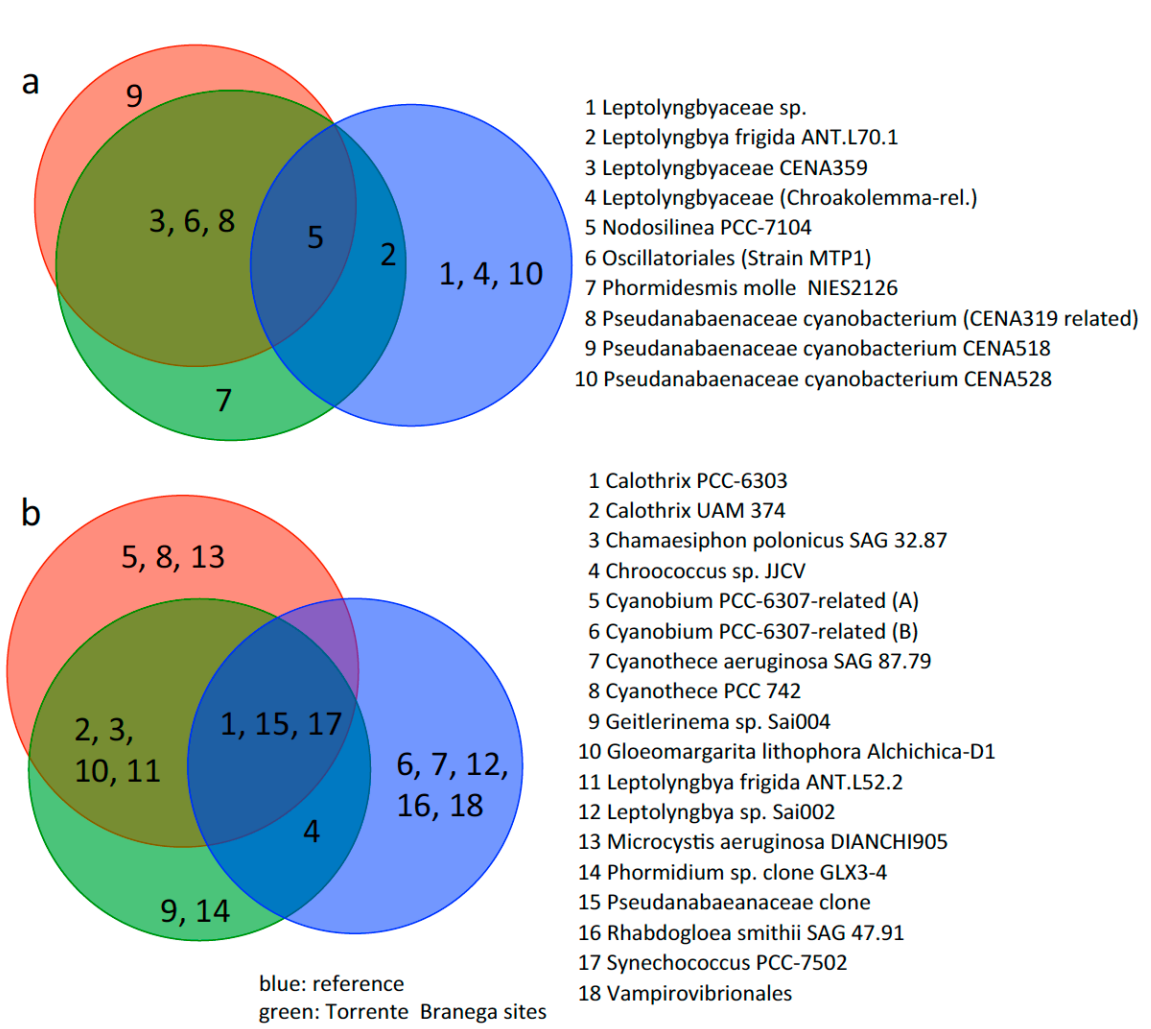
Venn diagrams for (**a**) high abundant (**a**) and minor (**b**) cyanobacterial groups in samples from alkaline springs and creek water, as indicated.

Venn diagrams illustrate the overlap in the cyanobacterial communities of both sites (Torrente Lerone, Torrente Branega), compared with the reference (Figure 8). In alkaline spring samples, few cyanobacterial groups are highly abundant (in this case, taxa represented by more than 2% of all OTUs in one or more of the samples; see Figure 5); they are different from the abundant groups detected in creek water sediment samples. The composition of the cyanobacterial communities in all alkaline spring samples is quite similar, with two dominating OTUs in most samples, related to the Leptolyngbyales strain CENA359 (up to 30% of all retrieved OTUs) and the Oscillatoriales strain MPT1 (up to 14%). Other frequent cyanobacteria are the Pseudanabenaceae cyanobacterium CENA518 and CENA319-related Phormidiaceae, being abundant just in some of the alkaline spring samples. A *Chroakolemma*-related Leptolyngbyaceae cyanobacterium and the Pseudanabaenaceae cyanobacterium CENA528 dominates the cyanobacterial community in creek water, whereas a *Leptolyngbya frigida* ANT.L70.1-related organism is present, in lower amounts, both in freshwater and in some of the alkaline spring samples. Another *Leptolyngbya*-related OTU (*Leptolyngbya frigida* ANT.L52.2) is abundant in all samples from alkaline springs. Among low abundant cyanobacterial taxa (Figure 6 and Figure 8b) a *Synechococcus* PCC-7502-related OTU was retrieved from all samples. This is the only taxon present in all alkaline spring samples, as well as creek water samples. Other taxa (*Calothrix* PCC-6303-related, Pseudanabaenaceae-rel.) are not present in all samples, but occur in some of the alkaline spring samples, and in creek water. Samples VE02-02.B and V04-01-BR1-1 exhibited a higher diversity among the rare cyanobacterial taxa than the other samples.

Other abundant bacterial phyla are Proteobacteria (28–38% of all OTUs), Bacteroidetes (15–25% of all OTUs), Planctomycetes (2–15%), and Verrucomicrobia (1–8%). In the following, some of the abundant genera are presented. Among other bacteria (Figure 5), a betaproteobacterial OTU related to *Hydrogenophaga* is of highest abundance in most of the samples, but is missing in the creek water sample. In a similar way, two Xanthomonadaceae, one identified as *Silanimonas*-related, are abundant in the alkaline springs, but absent in creek water. An Acetobacteraceae bacterium (related to *Rhodovarius* and *Sediminicoccus rosea*) is well present in all alkaline spring samples, but is also represented by few sequence reads in creek water. Two *Tabrizicola*-related OTUs are also present in all alkaline and creek water samples. Other, non-cyanobacterial taxa are either present in alkaline springs but not in creek water (e.g., Hyphomonadaceae, Rhizobiales A0839, *Pirellula*), or in creek water with low or no occurrence in alkaline springs (*Polymorphobacter*).

## 4. Discussion

Microbial ecosystems associated with serpentinization have been reported for seafloor hydrothermal chimneys (for instance [44,45]), alkaline springs on land [46,47,48], and wells drilled into serpentinizing rocks [25,49,50,51]. Systems exposed to sunlight, such as the alkaline springs and associated pools in this study, should be expected to develop stable microbial communities based on phototrophic primary producers. Four OTUs (A: Oscillatoriales/Strain MTP1-related, B: *Pseudanabaenaceae cyanobacterium* CENA518-related, C: *Leptolyngbyaceae* CENA359-related, D: *Pseudanabaenaceae cyanobacterium* CENA319-related) are most widespread in alkaline samples and are of highest abundance. Related taxa have been already described for alkaline water bodies, including a serpentinizing ecosystem (OTUs B, D) [47,52]. One was described as extremophilic (thermophilic, heavy metal resistant: OTU A from an enrichment culture of a sample obtained at a thermal source at Chalk Creek, Colorado [53]), or related to carbonate precipitation (OTU C) [54]. Two OTUs are exclusively retrieved from non-alkaline creek water (*Chroakolemma*-related *Leptolyngbyaceae*, *Pseudanabeaceae Cyanobacterium* CENA528). It may be surprising that the latter has been described as an isolate from Brazilian alkaline lakes [52], however, since the isolate is able to grow on a standard growth medium at pH 7.8 [55], it may not necessarily be an obligate alkaliphilic strain. Three OTUs (related to *Nodosilinea* PCC-7104, *Leptolyngbya frigida* ANT.L70.1, or *Phormidesmis molle* NIES2126) were retrieved from freshwater and alkaline spring sites, accounting for a broad ecological amplitude of these organisms.

A look at cyanobacterial OTUs being present in low abundance (less than 2%, Figure 6) revealed, in a similar way to the abundant taxa, differences between the alkaline and the creek community, but also some more shared taxa (Figure 8b). *Synechococcus* PCC-7502 is present in all samples, whereas *Leptolyngbya frigida* ANT.L52.2 just occurs in alkaline spring samples. Other serpentinizing springs may not necessarily host similar or identical cyanobacterial taxa. The dominant OTUs from an alkaline spring system within the Del Puerto ophiolite [56] are mainly *Leptolyngbya*-related OTUs without closer relationship to the OTUs gained in this study. This may be due to the fact that the cyanobacterial mats were retrieved from water bodes at pH 9, i.e., at considerably lower pH. From a serpentinizing spring site at the Chimaera ophiolite (Turkey), cyanobacterial OTUs in low amounts (up to 3.4% of all retrieved sequences) were obtained, with members of the Pseudanabaenales order at high abundance among cyanobacteria in all samples, which corresponds to samples from the Voltri sites. Generally, in all Chimaera seep sites, discharge is very low, and the formation of pools could not be observed. This may be a reason for the overall low abundance of cyanobacteria [57,58].

In all alkaline spring samples from Torrente Lerone and Torrente Branega, cyanobacterial filaments were either layered below consolidated carbonate crusts, and appeared as microstromatolite-like structures, or they were covered by dispersed, loosely-attached calcite particles (cf. [59]). In any case, cyanobacterial biofilms were not exposed at a surface, but below a calcite layer (Figure 1d,e), which suggests a similar lifestyle as known from endolithic communities (e.g., [60]), putatively in order to avoid exposure to highest light intensities at the sediment surface. This strategy has been first observed in hot deserts [61] and Antarctic dry valleys [62]. Since photoinhibition by ultraviolet and visible light affects cyanobacterial photosynthetic activity at high daylight intensities [63], endolithic lifestyles increase the fitness of these terrestrial cyanobacteria. Generally, patterns of colonization observed by eukaryotic algae, but also fungi, imply that endolithic lifestyles are widespread, if not inevitable, when the colonized surface is exposed to high light intensities [64]. Though the environments of the Voltri sampling sites are aquatic (with dry seasons in summer), the water layers up to decimeter range do not reduce photoinhibition by visible light, because absorption of visible light is negligible. Though calcite is a translucent mineral, a layer of small calcite particles will reduce the overall light intensity by scattering. In addition, calcite absorbs ultraviolet light with a maximum at 240 nm wavelength [65].

Cyanobacterial filaments were covered with smaller cells of rod-like bacterial morphotypes, which is indicative for a bacterial community that benefits from the cyanobacterial primary producers (Figure 4b, inset). It is not known which part of the microbial community interacts with the cyanobacterial filaments, but bacterial epibionts have been repeatedly observed and characterized in freshwater and marine filamentous cyanobacteria [66,67,68]. The organisms may benefit from the cyanobacterial metabolic products such as extracellular glutamic acid; nutrient flow from cyanobacteria to heterotrophic bacteria in freshwater environment has been shown for *Oscillatoria redekei* (*Limnothrix redekei*) and isolates of heterotrophic bacteria [69]. It should not be neglected that cyanobacterial sheath exopolysaccharides, composed of well-biodegradable monosaccharides (cf. [70]), as well as decaying cells, may putatively feed the heterotrophic bacteria.

Among proteobacterial groups, a *Hydrogenophaga*-related OTU was most abundant in the alkaline springs. A subset of *Hydrogenophaga*-related strains comprises typical representatives of the highly alkaline and calcium-rich serpentinizing springs [45,46,71], including the Voltri spring sites [23]. These are also abundant in other alkaline water bodies [72]. *Hydrogenophaga* isolates are facultatively autotrophic bacteria, utilizing a variety of carbohydrates [73,74], but also hydrogen and carbon monoxide; carbon dioxide fixation is conducted via the Calvin cycle [71]. In addition, mixotrophic growth on organic and inorganic substrates at the same time is possible, which may reflect the adaptation to the spring habitat where the availability of hydrogen gas may be rather low, in particular, in some distance to the spring site (cf. [15]), but input of organics from primary producers (as shown here) may provide substrates for a heterotrophic lifestyle.

A proteobacterial OTU related to *Silanimonas* is widespread in all sampled spring sites, and absent in creek water. *Silanimonas* strains are aerobic heterotrophic and alkaliphilic organisms [25,75]. Other Proteobacteria abundant in alkaline spring samples are *Xanthomonadaceae* and *Hyphomonadaceae*-related OTUs that cannot be further taxonomically resolved, but are representatives of oligotrophic freshwater bacteria, being widespread and not necessarily alkaliphilic. Two *Tabrizicola*-related OTUs are, though of higher abundance in many (but not all) of the spring sites, also present in freshwater. The type strain (*Tabrizicola aquatica* [76]) grows heterotrophically on various carbohydrates, and chemolithoautotrophically on reduced sulfur compounds.

One abundant retrieved *Acetobacteriaceae* OTU may be unexpected, because it belongs to a group with many acidophilic members, adapted to rather nutrient-rich habitats [77]. Genera related to these OTUs, however, are isolated from aquatic environments. The Acetobacteriaceae member *Sediminicoccus rosea* was isolated from a lake sediment [78] and *Rhodovarius*-strains are freshwater isolates as well [79]. One of the retrieved OTUs is also related to one detected in an Antarctic freshwater environment in a cyanobacterial mat covering a moss pillar [80]. None of these isolates or OTUs, however, were adapted to high-alkaline pH.

Some taxa within the Bacteroidetes take benefit from the alkaline spring site habitat. Many OTUs related to this group are also abundant in freshwater samples, but in low counts. Bacteroidetes listed in Figure 5 are just associated with the alkaline springs. One important reason may be that members of the group take benefit from the presence of cyanobacteria as their primary resource of organic nutrients (cf. [81]).

Interestingly, the two dominant *Luteolibacter* OTUs are present either in the control sample or in alkaline spring sites. The related OTUs SINN488 and FGL7S_B75 were, according to NCBI database entries, retrieved from lake habitats [82,83]. Another verrucomicrobial OTU (*Lacunisphaera*-related [84]), occurs in the same samples from alkaline sites as *Luteolibacter.*

Taking the data together, the cyanobacterial mats of the serpentinizing springs are important primary producers, and are colonized by microorganisms known from other highly-alkaline water bodies; some few abundant representatives of them are also typical for other terrestrial serpentinizing springs. Many proteobacterial and Bacteroidetes-related OTUs may benefit from the organic input by the cyanobacterial mat.

An interaction between microbial cells and the carbonate precipitates of the spring sites is obvious and inevitable. Light and scanning electron microscopic analysis revealed the presence of microbial cells (putatively cyanobacterial sheaths) embedded in calcite fabric. Surfaces of these carbonates show a typical honeycomb structure, which often occurs at spring outflows (Figure 1a,b). The otherwise-white calcite shows a distinct brown stain due to impregnation with iron hydroxides. The light-yellow calcites from some Torrente Lerone springs show a periodic zonation of 10–20 µm in thickness and are reminiscent of microstromatolites (cf. [5]). Putatively, the zonation is caused by different contents of dispersed intracrystalline organic material, representing a mineralized polysaccharide-rich exopolymeric matrix of microbial mat remains. This periodicity may reflect seasonal changes in temperature and, hence, variation in evaporation rate of the outflowing spring water. In many other cases, no zonation is visible (cf. Figure 3a), but entrapped organics and prokaryotic cell shapes reveal that a bacterial biofilm must have been present and was rapidly encased by precipitation of calcites.

Isotopic values obtained from representative sampling sites turned out to be considerably variable. Torrente Lerone carbonates exhibit δ^13^C_CaCO__3_ values between −24% and −11%; δ^18^O range from −19% to −9%. In Torrente Branega, the measured δ^13^C values are close to 0%, up to +0.6%. δ^18^O values range between −20% and −26%. Generally, carbon isotope fractionation depends on two relevant processes, i.e., the equilibrium exchange reaction between atmospheric CO_2_, dissolved bicarbonate and precipitated carbonate (δ^13^C in solid carbonate is enriched), and biological processes like methanogenesis, anaerobic oxidation of methane, or photosynthesis. The alkaline fluids seeping out of the serpentinite mountain massif are enriched in light carbon, possibly caused by anaerobic methane oxidation [15]. The surplus of isotopically-light dissolved carbonate ions (putatively generated by anaerobic methane oxidation in deep aquifers) precipitate fast to carbonates, close to the outflow of the spring. In increasing distance to the outflow, atmospheric carbon dioxide with a δ^13^C of c. −8.5% [85] is equilibrating with remaining dissolved carbonate ions, and less depleted carbonates precipitate. This may explain the large range of δ^13^C values in the Torrente Lerone carbonates [19]. An influence of photosynthetic processes of cyanobacteria, which also retract ^12^C from the carbon pool, cannot be excluded. Another travertine-type carbonate buildup taken from the Torrente Branega site differs from the Lerone locality. The measured δ^13^C values of carbonates are close to 0%; few exhibit +1.5% slightly heavier values, eventually caused by photosynthetic processes, as mentioned above. Interestingly, the δ^13^C values are in the same range as those of dissolved inorganic carbon (DIC) of seawater. It may be speculated that either the spring fluids reflect an original seawater signal or that the originally isotopically light carbon in meteoric water (c. δ^13^C_CO__2atm_ −8.5%, see above) was depleted in ^12^C during, e.g., methanogenesis within the aquifers of the Voltri Massif. Consequently, the remaining fluid became a less depleted “seawater” signal. Methanogenic and methanotrophic archaea, both involved in carbon isotope fractionation, were already detected in spring water from the Voltri Massif. Several studies [15,17,19] reported similar δ^13^C and δ^18^O values in carbonates as seen in the Torrente Lerone carbonate buildups, whereas our data from Torrente Branega considerably differ from these findings. Various types of water feeding the springs may explain this. A substantial influence of photosynthetic processes in carbonate fractionation could not be observed.

## 5. Conclusions

A specific deep biosphere methane- and hydrogen-metabolizing community may inhabit deep aquifers in serpentinizing spring sites. Close to outflows and in alkaline pools at spring sites, the microbial community has changed: few cyanobacterial OTUs (a Leptolyngbyales-related strain and an Oscillatoriales-related strain with up to 30% and up to 14% of all retrieved OTUs in single samples, respectively) are by far the most abundant bacteria forming mats associated with precipitated carbonates. They are nearly absent in reference samples from creek water. A fast (chemical, non-biological) precipitation of carbonates is influenced by cyanobacterial exopolymeric substances, which becomes obvious when consolidated microstromatolite-like carbonate buildups are formed. There is, however, no considerable difference in microbial communities for more or less consolidated carbonates. Though the spring site water body is oligotrophic, and cyanobacterial layers are rather inconspicuous, they may develop undisturbed by grazers, due to the ultra-high pH, and provide a small, but continuous, nutrient resource for a bacterial community, associated with the cyanobacterial filaments.

## Figures and Tables

**Table 1:** Selected water chemistry parameters of the sampling sites (see Table S1 for a complete dataset from alkaline springs), Ler: Torrente Lerone samples; Br: Torrente Branega samples.

Parameter	Ler	Br	Creek Water Reference (Br)
pH	11.2–11.3	11.2–11.5	8.45
Eh	−60 mV	+180–+230 mV	+370 mV
Ca^2+^	34–43 mg L^−1^	33–47 mg L^−1^	19 mg L^−1^
Na^+^	8–10 mg L^−1^	23–25 mg L^−1^	14 mg L^−1^
K^+^	0.7–3.1 mg L^−1^	2.8–3.1 mg L^−1^	1.3 mg L^−1^
SO_4_^2−^	2.0–2.4 mg L^−1^	0.1–0.5 mg L^−1^	5.9 mg L^−1^
NO_3_^−^	0.005–0.014 mg L^−1^	0.005–0.030 mg L^−1^	1.5 mg L^−1^
NH_4_^+^	0.03–0.04 mg L^−1^	0.03–0.12 mg L^−1^	0.01 mg L^−1^
H_2_S	0.2–0.7 mg L^−1^	0.006–0.05 mg L^−1^	n/a

## Supplementary Materials

The following are available online at https://www.mdpi.com/2076-2607/9/1/62/s1, Figure S1: Stable δ^13^C_CaCO__3_ and δ^18^O_CaCO__3_ isotopic records of samples from carbonate buildups (Torrente Lerone/Torrente Branega sampling sites as indicated) and samples from cemented carbonate fractures within ophicalcites NE of Bonassola/La Spezia. Figure S2: Rarefaction curve depicting all samples as indicated. Table S1: Extended table of all hydrochemical data taken from alkaline springs and from creek water as indicated.
