# Supplementary material for: Cyanobacterial Mats in Calcite-Precipitating Serpentinite-Hosted Alkaline Springs of the Voltri Massif, Italy
Source: Microorganisms. 2020 Dec 29;9(1):62. doi: 10.3390/microorganisms9010062 (PMC7824716; doi:10.3390/microorganisms9010062)
Supplement: Supplementary file 1 [file microorganisms-09-00062-s001.zip › microorganisms-982865-s/microorganisms-982865_Figures_S1_S2_Cyanobacterial mats in calcite precipitating serpentinite-hosted alkaline springs of the Voltri Massif_review_1.pdf]

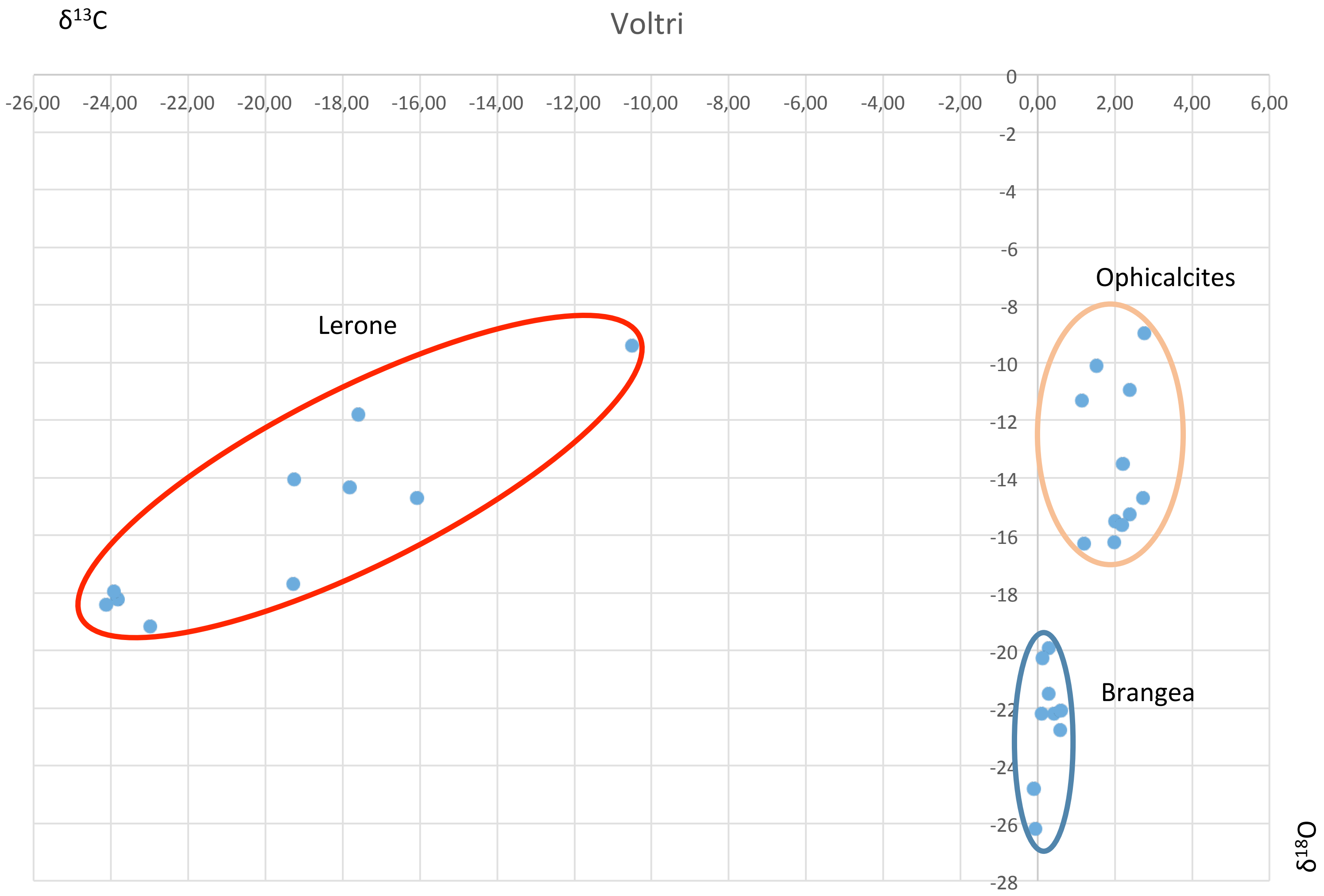

Figure S1: Stable delta$^{13}C_{CaCO3}$ and delta$^{18}O_{CaCO3}$ isotopic records of samples from carbonate buildups (Torrente Lerone/Torrente Branega sampling sites as indicated) and samples from cemented carbonate fractures within ophicalcites NE of Bonassola/La Spezia.

Fig. 1 - supplement

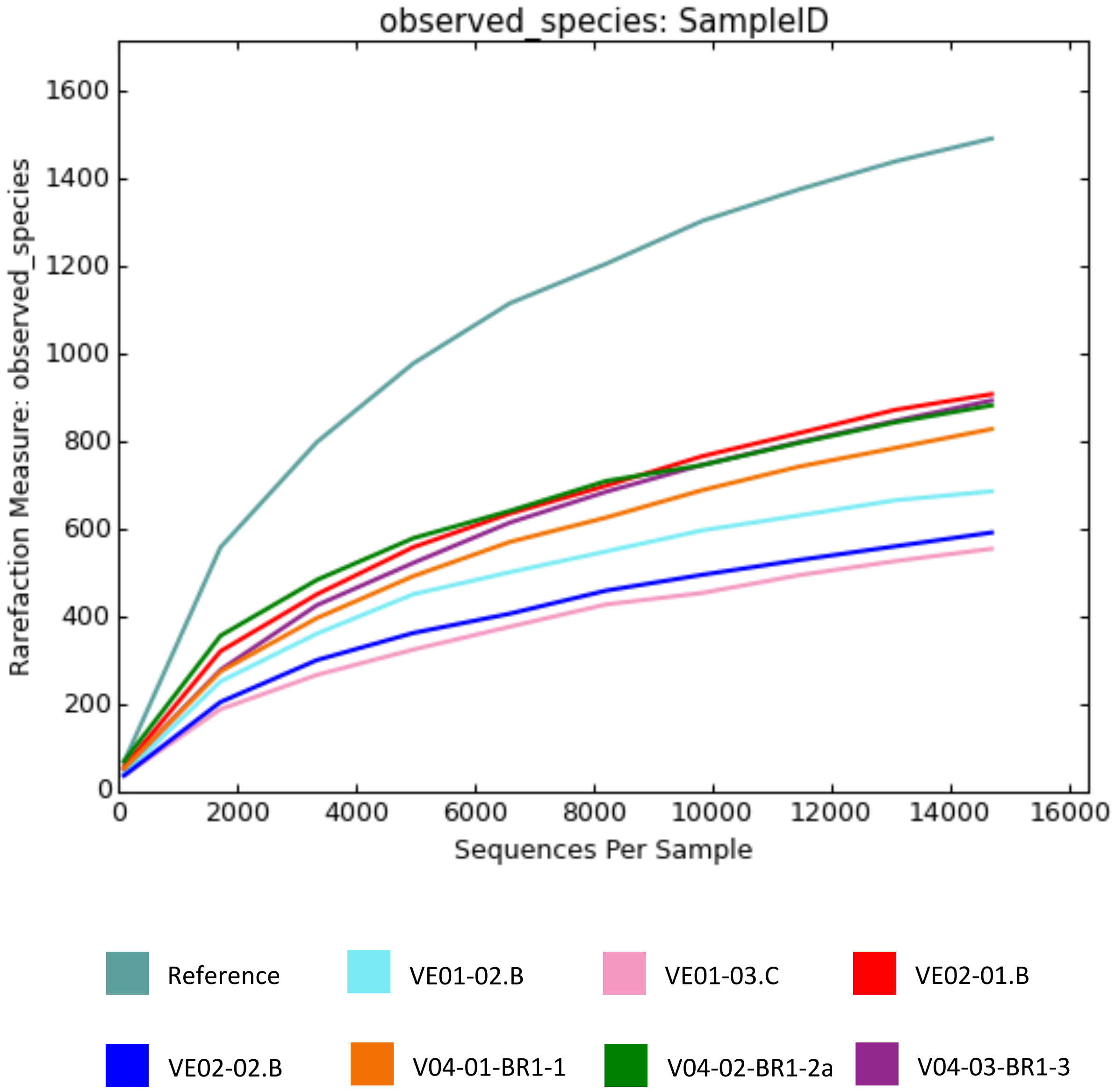

Figure S2: Rarefaction curve depicting all samples as indicated.
